# coMET: visualisation of regional epigenome-wide association scan results and DNA co-methylation patterns

**DOI:** 10.1186/s12859-015-0568-2

**Published:** 2015-04-28

**Authors:** Tiphaine C Martin, Idil Yet, Pei-Chien Tsai, Jordana T Bell

**Affiliations:** Department of Twin Research and Genetic Epidemiology, St Thomas’ Hospital Campus, King’s College London, Westminster Bridge Road, London, UK

**Keywords:** Epigenome-wide association scan, EWAS, DNA methylation, Co-methylation, Visualisation, Gene expression, Functional annotation, Bioconductor

## Abstract

**Background:**

Epigenome-wide association scans (EWAS) are an increasingly powerful and widely-used approach to assess the role of epigenetic variation in human complex traits. However, this rapidly emerging field lacks dedicated visualisation tools that can display features specific to epigenetic datasets.

**Result:**

We developed coMET, an R package and online tool for visualisation of EWAS results in a genomic region of interest. coMET generates a regional plot of epigenetic-phenotype association results and the estimated DNA methylation correlation between CpG sites (co-methylation), with further options to visualise genomic annotations based on ENCODE data, gene tracks, reference CpG-sites, and user-defined features. The tool can be used to display phenotype association signals and correlation patterns of microarray or sequencing-based DNA methylation data, such as Illumina Infinium 450k, WGBS, or MeDIP-seq, as well as other types of genomic data, such as gene expression profiles. The software is available as a user-friendly online tool from http://epigen.kcl.ac.uk/cometand as an R Bioconductor package. Source code, examples, and full documentation are also available from GitHub.

**Conclusion:**

Our new software allows visualisation of EWAS results with functional genomic annotations and with estimation of co-methylation patterns. coMET is available to a wide audience as an online tool and R package, and can be a valuable resource to interpret results in the fast growing field of epigenetics. The software is designed for epigenetic data, but can also be applied to genomic and functional genomic datasets in any species.

## Background

Epigenome-wide association studies (EWAS) systematically test for association between DNA methylation variation and human complex traits [1,2]. Recent EWAS have identified differentially methylated regions (DMRs) and variably methylated regions (VMRs) for multiple phenotypes, diseases, and environmental exposures (for example, [3-7]) and many on-going efforts are currently underway. A major challenge lies in interpreting EWAS signals. One problem is defining the exact region of association, for example, distinguishing between a single differentially methylation position (DMP) and a differentially methylated region (DMR) containing multiple DMPs. DNA methylation levels at nearby CpG-sites (within 2kb apart) can be highly correlated [8,9]. Analyzing clusters of co-methylated CpG sites may be more informative than single-CpG analysis [9], yet from a functional perspective identifying specific DMP(s) with potential molecular consequences is critical. A number of recent packages including Bumphunter [10], Minfi [11], ChAMP [12], A-clustering [13], and RnBeads [14] can identify DMRs, but none of these methods allow the visualization of the correlation between DMPs within a DMR. On the other hand, several R packages, including snp.plotter [15] and LocusZoom [16] have been developed to visualise GWAS association results and linkage disequilibrium (LD) patterns, but none exists for EWAS. At present, EWAS datasets typically consist of quantitative levels of DNA methylation at different sites or regions, where each sample represents a population of cells from an individual. These data are therefore unsuitable for standard LD plots and would benefit from dedicated user-friendly methylation plotting tools.

EWAS findings can provide mechanistic insights into disease susceptibility or progression, and should be explored in functional genomic context. A comparison across multiple layers of epigenetic marks and chromatin domains can help define the functional context of a genomic region. Several R packages, such as Gviz [17], trackViewer [18], ggbio [19], GenomeGraphs [20], or methyAnalysis [21], can help us to visually explore different annotation tracks in genomic regions. However, most of these packages do not have options to concurrently visualise phenotype-association results or co-methylation patterns at CpG-sites or regions.

We have developed coMET, an R package and web-based tool to generate regional plots of EWAS data and results. coMET can be applied to visualise regional EWAS results from analyses of both single-CpG and region-based datapoints, for example using microarray-based technologies (such as Illumina 450k) or sequencing-based methods (such as whole genome bisulfite sequencing (WGBS) or DNA methylation capture by immuno-precipitation followed by sequencing (MeDIP-seq)), and to compute and visualise also the correlations between the CpG-sites or regions. The web-service [22] allows users to run a pre-formatted version of coMET.

## Implementation

coMET is implemented in R to produce a multi-panel plot to visualise EWAS results, genomic annotations, and to estimate and plot DNA co-methylation patterns. The structure of the plots builds on snp.plotter [15], with extensions to incorporate genomic annotation tracks and customized functions. Plots can be generated in PDF or EPS format. coMET is available as an R package or as a web-service.

### coMET R package

The coMET R package is available for download from Bioconductor [23] or online from GitHub [24]. The package includes source code, two sample datasets, documentation and vignette. Analysis requires R version 3.1.1 or higher and an active internet connection to enable direct download of up to date annotation tracks from Ensembl mart databases [25] and UCSC [26]. The coMET R Bioconductor package has currently two main functions: ’comet.web’ and ’comet’. The function ’comet.web’ generates output plots with the predefined genomic annotation track settings used for the web-service and the function ’comet’ generates output plots with customised annotation tracks defined by the user.

### coMET web-service

The coMET website [22] allows users to run a pre-formatted version of coMET. The web-service is developed in Shiny [27] and can be installed locally on a machine running R version 3.1.1 or higher, Bioconductor version 3.1 or higher and Shiny. It requires at least 4GB of memory and at least 10GB of available disk space. The web-service also requires an active internet connection to download up to date annotation tracks.

### Data input

**Figure 1:**
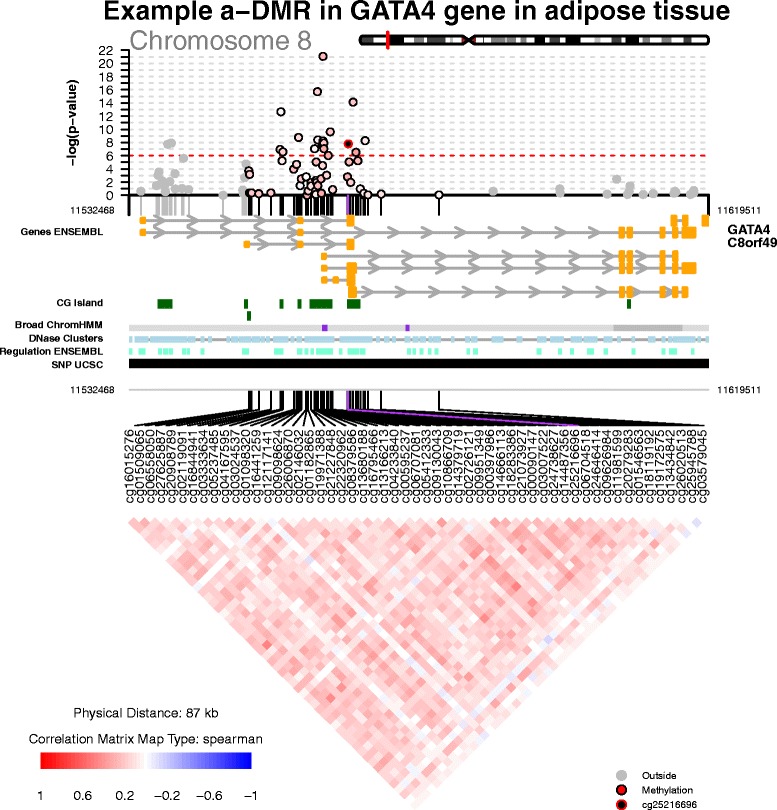
Regional plot of age-EWAS results and co-methylation patterns at the *GATA4* gene in adipose tissue. Methylation data were obtained from previously published publicly available Illumina 450k profiles from 648 samples in adipose tissue [28].

Data input for coMET includes a data file or matrix describing EWAS results at the DNA methylation CpG-sites or genomic regions to visualise ("mydata.file" and "mydata.type"), a data file or matrix describing the co-methylation dataset ("cormatrix.file" and "cormatrix.type"), and a configuration file containing the plot parameters ("config.file"). Currently, coMET can visualise the correlation of a maximum of 120 pre-defined features on the plot, due to limitations on the size of the plot. Optional input files can also be uploaded to include association P-values from a larger genomic region of interest in the upper plot or to include them as user-defined annotation tracks in the middle panel, but the lower panel is limited to visualizing only up to 120 CpG-sites or regions which can represent a subset of the larger genomic region in the top panel if required (see example in Figure 1).

## Results and discussion

coMET generates a multi-panel plot to visualise EWAS results, co-methylation patterns, and annotation tracks in a genomic region of interest. A coMET figure (Figure 1) includes three components: (1) the upper plot shows the strength and extent of EWAS association signal; (2) the middle panel provides customized annotation tracks; and (3) the lower panel shows the co-methylation between selected CpG sites in the genomic region.

### EWAS signal

The top panel of the coMET plot shows EWAS phenotype association P-values on a -log10 scale according to chromosomal position. The user can specify the region of interest in the input dataset and zoom in/out to view a subset of the region. A reference CpG-site can be highlighted in the association plot, using user-specified colours. The association plot can also optionally denote the direction of phenotype-association using colour-coded symbols. If region-based rather than single-CpG datapoints are visualised the plotting symbols can also denote the size of the regional unit of analysis.

### Annotation tracks

The middle panel includes optional genomic annotation tracks, for example, functional annotations from Ensembl, ENCODE and UCSC for any species and versions available such as hg38, hg19 or GRCm38. By default, the pre-formatted version of coMET (*‘*comet.web’) includes six optional annotation tracks: genes or transcripts (Ensembl), CpG islands (UCSC), Broad ChromHMM domains (UCSC), DNaseI clusters (UCSC), Ensembl regulation tracks, and SNPs (UCSC). These tracks are obtained directly from the revelant online server or data repository (Ensembl BioMart or UCSC tracks) at the time of analysis. This allows for visualisation of up to date functional annotation data, but the analysis requires an active internet connection. In addition to the optional pre-defined tracks, user-defined annotation and data tracks can also be included in a format accepted by Gviz. Altogether, up to 6 annotation tracks can be viewed in the basic version of the package. The generic version of coMET (‘*comet*’) can visualise lists of customized annotation and data tracks using Gviz [17], ggbio [19], and trackViewer [18].

### Co-methylation

The lower panel represents the correlation in DNA methylation levels between selected CpG-sites in the genomic region, or co-methylation. The correlation matrix and the significant of correlations are calculated based on user-provided DNA methylation values (e.g. beta values for the Illumina 450k array) for selected CpG-sites or regions, the selected correlation method (Spearman, Pearson, Kendall), and the selected alpha level for the confidence interval (e.g. alpha=0.05 for 95% CI). A user-provided correlation matrix can also be used. The colour scheme of the heatmap represents the correlation scale (for example, from -1 (blue) to 1 (red) in Figure 1) and can be reflected in the top association panel of the coMET plot with respect to correlation to the user-defined reference CpG site.

### Examples

To show the functionality of coMET, we explored previously published Illumina 450k DNA methylation profiles from adipose tissue in 648 individuals with available age at biopsy [28]. We first performed an EWAS of chronological age and found strong age association with the previously identified age differentially methylation region (a-DMR) in the *GATA4* gene, which is an a-DMR in whole blood, muscle, kidney, and brain samples [29,30]. Figure 1 shows age association results at 95 CpG sites (upper plot), with 6 default annotation tracks, and estimated co-methylation patterns at 55 selected CpG sites (lower panel) in the *GATA4* gene on chromosome 8. The results are shown with respect to reference CpG site cg25216696, which is the most associated a-DMP in our data and in previously published whole blood datasets. These findings show for the first time that the *GATA4* a-DMR is present in adipose tissue.

Recently, Richmond et al. [31] also used the online version of coMET to visualise differential methylation and co-methylation patterns of 7 genomic regions using Illumina 450k data.

## Conclusion

coMET is a user-friendly R-package and online tool that allows for quick and flexible visualisation of EWAS results, co-methylation patterns, and functional annotation. The software is designed for epigenetic data, but can also be applied to genomic and functional genomic datasets in any species.

## Availability and requirements

**Project name**: coMET **Project home page**: http://epigen.kcl.ac.uk/comet**Operating system(s)**: Platform independent **Programming language**: R 3.1.1 or higher **Other requirements**: Bioconductor 3.1 or higher, Shiny **License**: GNU GPL 2 or higher **Any restrictions to use by non-academics**: none
